# The Effect of Air Pollution on the Occurrence of Nonspecific Conjunctivitis

**DOI:** 10.1155/2016/3628762

**Published:** 2016-05-30

**Authors:** Zhiwei Li, Xiaoyan Bian, Jianguang Yin, Xiaoli Zhang, Guoying Mu

**Affiliations:** ^1^Department of Ophthalmology, Shandong Provincial Hospital Affiliated to Shandong University, Jinan 250021, China; ^2^Baotou Chaoju Eye Hospital, Baotou 014000, China; ^3^State Grid Shandong Electric Power Research Institute, Jinan 250021, China

## Abstract

*Purpose.* To investigate the short-term effect of air pollution on occurrence of nonspecific conjunctivitis.* Methods.* Data were collected from outpatient visits from cases with conjunctivitis over a period of one year. Regression analysis was performed to evaluate the relationship between the number of outpatient visits and the air quality and the lag effect of air quality on conjunctivitis occurrence.* Results.* The air quality index on the day of presentation (*P* = 0.023), one day before presentation (*P* = 0.049), and two days before presentation day (*P* = 0.050) had a positive relation with outpatient visits for conjunctivitis. The air quality index (*P* = 0.001) and outpatient visits number per day (*P* = 0.013) in autumn and winter (October to March) were significantly higher than those in spring (April) and summer (September).* Conclusions.* The air quality index within two days before presentation affected the probability of attending the outpatient clinic for nonspecific conjunctivitis. High number of cases can be expected in colder season.

## 1. Introduction

Air pollution is a risk factor for various diseases including eye irritation, respiratory infections, and heart disease [1–3]. Conjunctiva is sensitive to environmental particles considering the direct contact of conjunctiva with the outside environment [4]. Conjunctiva protects the ocular from outsides deleterious agents, helps lubricate the eye by producing mucus and tears, and contributes to the immune balance of ocular surface. The importance of conjunctiva and a high prevalence of conjunctivitis merit an investigation on the effect of air pollutant on conjunctivitis.

The environmental pollution, especially the air quality, has deteriorated in the past decades in China mainly due to the rapid industrialization in the country [5]. The maximal air quality index can reach above 500 in some parts of China. Overall, no more than 5 cities among the 500 largest cities of China meet the air quality guidelines recommended by the World Health Organization. Recently, seven cities in China were ranked among the 10 most polluted cities in the world [6]. The current study aims to evaluate the effect of air pollution on the occurrence of nonspecific conjunctivitis through analyzing the patients diagnosed as nonspecific conjunctivitis in Jinan city and the air pollution level of Jinan city.

## 2. Methods

Data was collected from two eye centers in Jinan city: central area and east area of Shandong Provincial Hospital, Shandong University. Patients presenting to the outpatients clinic between June 2014 and May 2015 with symptoms and signs of nonspecific conjunctivitis were included. Outpatient visits for nonspecific conjunctivitis were selected according to a previously published report [7] and the International Classification of Diseases (ICD-9) diagnostic codes. The following codes were included: 372.00, 372.01, 372.10, 372.11, 372.20, and 372.30 (for nonspecific acute conjunctivitis, serious conjunctivitis except viral infection, chronic conjunctivitis, simple chronic conjunctivitis, blepharoconjunctivitis, and other undefined conjunctivitis, resp.). The following cases were excluded: patients with other ocular diseases including corneal abnormalities, conjunctivitis before the initiation of the study, xerophthalmia, and systemic immune disease.

Air pollution data was harvested from the State Environmental Protection Administration of China and expressed as air quality index (AQI). The AQI was composed by the index of particulate matter (PM_10_ and PM_2.5_), nitrogen dioxide (NO_2_), sulfur dioxide (SO_2_), ozone (O_3_), and carbon monoxide (CO).

The linear regression analysis was used to evaluate the relationship between number of clinic visits per day and AQI from the same day up to 4 prior days. The AQI on presenting day was expressed as AQI_0_. The AQI within 1 day, 2 days, 3 days, and 4 days were calculated as the mean of the AQI on presenting day and 1 day, 2 days, 3 days, and 4 days prior to presentation and were expressed as AQI_1_, AQI_2_, AQI_3_, and AQI_4_. Statistical analysis was performed with SPSS (version 16.0 for Windows). *P* < 0.05 was considered as statistically significant.

## 3. Results

**Figure 1:**
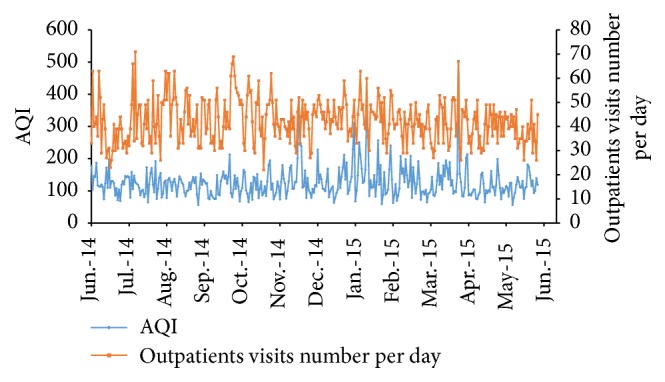
A total of 15373 patients were enrolled in this study from June 2014 to May 2015, and the AQI was recorded within same interval. The average patients number per day and AQI were 42 (22–71) and 125 (56–500), respectively.

A total of 15373 patients living in the air-quality-monitoring area of Jinan city were enrolled in this study. The average number of patients with nonspecific conjunctivitis per day was 42 (22–71), and the average AQI was 125 (56–500) (Figure 1).

**Figure 2:**
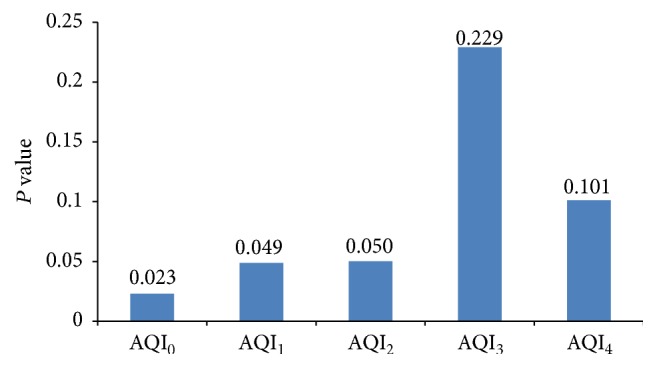
The patients number per day has positive relation with AQI_0_ (*P* = 0.023), AQI_1_ (*P* = 0.049), and AQI_2_ (*P* = 0.050), but not AQI_3_ (*P* = 0.229) and AQI_4_ (*P* = 0.101).

The AQI_0_ (*P* = 0.023), AQI_1_ (*P* = 0.049), and AQI_2_ (*P* = 0.050) had a positive relation with the number of patients per day (Figure 2). However, the AQI_3_ (*P* = 0.229) and AQI_4_ (*P* = 0.101) did not have a significant relation with patient numbers per day (Figure 2). The AQI (*P* = 0.001) as well as the number of patients per day (*P* = 0.013) in autumn and winter (October to March) was higher compared to that in spring and summer (April and September).

## 4. Discussion

In the present study, the AQI was harvested from 15 areas of Jinan district covering 3000 km^2^ and 4 million people. Previous studies have demonstrated the effect of air pollution on respiratory disorders [8, 9]. A similar reaction to exogenous stimuli between conjunctival mucosa and respiratory mucosa has been proposed in the past [10, 11]. Chang et al. [7] reported a positive relation between air pollution and outpatient visits for nonspecific conjunctivitis in Taiwan area. The different components of air pollutants have different effects on the occurrence of conjunctivitis [7]. In present study, we reported that the occurrence of conjunctivitis has positive relation with the AQI on presenting day and the AQI within one day before the day of presentation. A limitation of our study is that we did not investigate the effect of different components of pollutants on causation of conjunctivitis. Present study observed a variety of conjunctivitis types within ICD-9 code but did not predefine various forms of infections and allergic or physiological changes in tear film disorders except with the ICD codes. More study should be done to elucidate the correlation between these various types of conjunctivitis and the various air quality measurements that were monitored.

Present study revealed that the AQI in autumn and winter is higher than that in spring and summer. The same trend was observed in the number of outpatient visits. The effect of temperature and humidity on conjunctivitis should also be considered besides AQI. A high AQI in autumn and winter in Jinan may be due to more coal consumption for heating, use of firecrackers consumption from spring festival to lantern festival, and a more difficult spread of pollutants due to low temperature.

This study was carried out in an area with heavy air pollution, in which a variety of health disorders are related to pollutants. Although present study has revealed a relation between air pollution and conjunctivitis, more detailed investigations should be carried out to elucidate the effect of age and sex on the ophthalmic response to pollutant and the clinical treatment. Furthermore, the relationship between conjunctivitis and dry eye [12, 13] merits the investigation of effect of air pollution on dry eye and other more severe ophthalmic disorders related to dry eyes dry eye, such as microbial keratitis [14] and the decline in quality of life [15].
